# Evolving trends and persistent challenges in chronic left ventricular heart failure management: insights from a 9-year analysis of in-hospital outcomes in Germany

**DOI:** 10.1007/s00392-025-02679-4

**Published:** 2025-05-26

**Authors:** Anastasia Janina Hobbach, Jannik Feld, Jürgen Reinhard Sindermann, Holger Reinecke

**Affiliations:** 1 Department of Cardiology I, Coronary, Peripheral Vascular Disease and Heart Failure, University Hospital Münster, Albert-Schweizer Campus 1, 48149 Münster, Germany; 2 Institute of Biostatistics and Clinical Research, University of Münster, Schmeddingstraße 56, 48149 Münster, Germany

**Keywords:** Heart failure, Hospitalization trends, In-hospital mortality, Healthcare costs, German healthcare system, COVID-19

## Abstract

**Aims:**

Chronic left ventricular heart failure (CLHF) represents a significant public health challenge globally, marked by high morbidity and mortality. CLHF is a leading cause of hospitalization, placing considerable strain on healthcare systems. This study aims to analyze in-hospital outcomes for CLHF patients in Germany over nine years (2014–2022), examining trends in morbidity, mortality, healthcare costs, and complications.

**Methods and results:**

Data were sourced from the Federal Statistical Office (DESTATIS), covering 2,616,462 inpatient CLHF cases (ICD-10-GM I50.11-I50.14) in Germany from 2014 to 2022. CLHF hospitalizations increased from 288,019 in 2014 to 311,782 in 2019, then declined in 2020 but started again to rise from 2021 to 2022. The proportion of patients with advanced CLHF (NYHA stage IV) decreased slightly from 54.09% in 2014 to 50.36% in 2020 (*p* < 0.0001). Notable comorbidity trends included rising rates of atrial fibrillation and chronic kidney disease. Complications such as cardiogenic shock and acute kidney injury increased over time (both *p* < 0.0001). Despite a decrease in mortality rates from 2014 to 2019 (*p* < 0.0001), in-hospital mortality rose to 8.84% in 2022 (*p* < 0.0001). Median revenue volume and hospitalization duration showed a pre-pandemic increase but declined in 2020.

**Conclusion:**

The study highlights a complex interplay of factors affecting CLHF management in Germany, as a representative example for a high-income country. While advancements in treatment have improved some outcomes, the COVID-19 pandemic significantly impacted hospitalization trends and in-hospital mortality. The findings underscore the need for ongoing improvements in CLHF management and resilient healthcare strategies to address future challenges.

**Graphical abstract:**

Overview of CLHF hospitalizations in Germany (2014–2022). Key findings include an 8.3% increase in hospitalizations from 2014 to 2019, followed by a temporary drop during the COVID-19 pandemic. Despite this, CLHF-related mortality peaked in 2022, highlighting the ongoing burden of severe cases. Rising costs and complications, alongside increasing comorbidities, emphasize the need for strengthened CLHF management and a resilient healthcare system. AF: atrial fibrillation, CLHF: Chronic left ventricular heart failure; CKD: chronic kidney disease, NYHA: New York Heart Association, Tsd: thousands.

**Figure:**
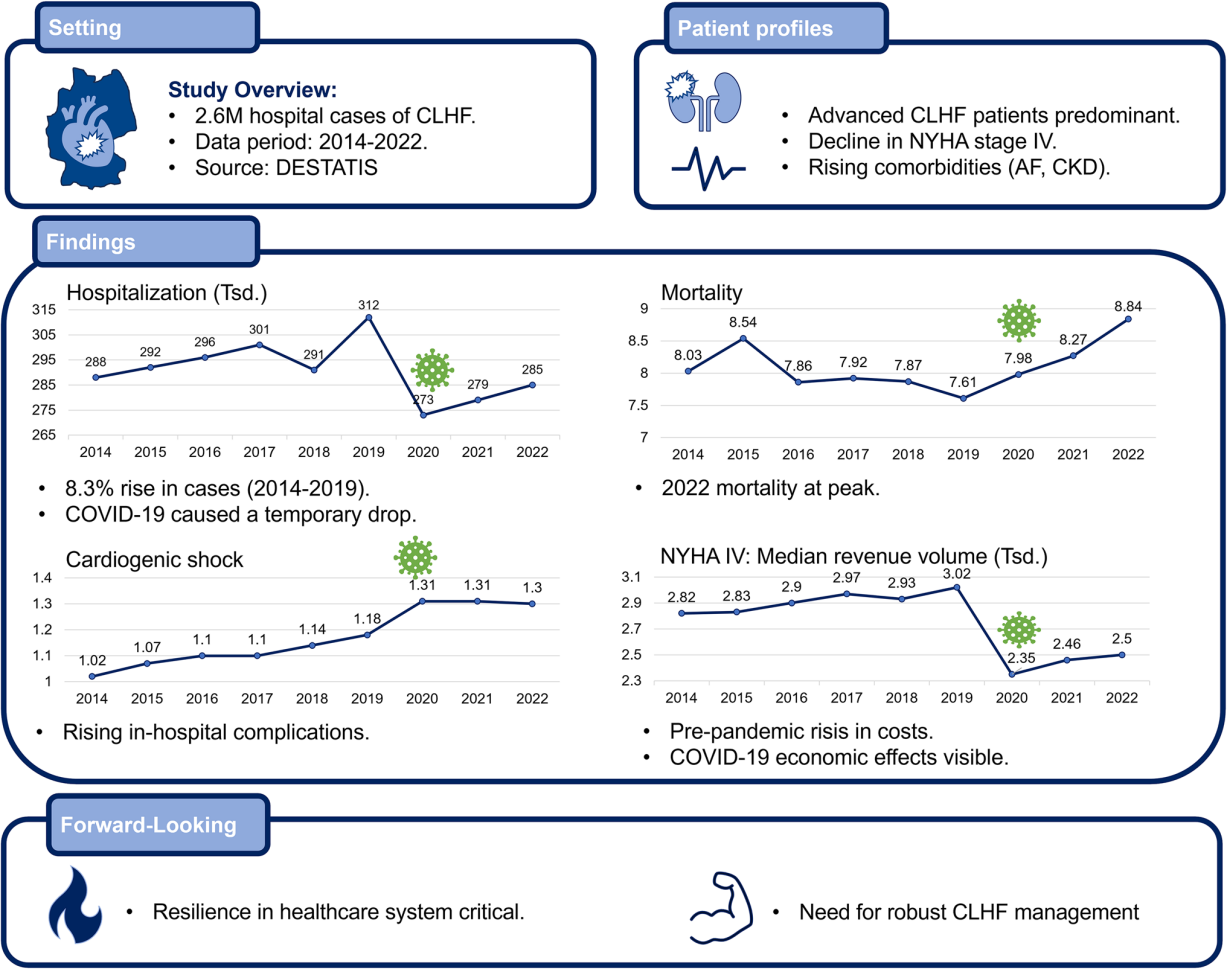

**Supplementary Information:**

The online version contains supplementary material available at 10.1007/s00392-025-02679-4.

## Introduction

Chronic left ventricular heart failure (CLHF) remains a major public health challenge worldwide, with significant morbidity and mortality rates [1]. In Germany, it is since a decade the single leading causes of hospitalization among adults, exerting substantial pressure on the healthcare system [2]. Despite advances in medical treatments and interventions, the burden of CLHF continues to escalate, driven by an ageing population and the rising prevalence of comorbidities [1]. The present study aims to provide a comprehensive analysis of in-hospital outcomes for patients with CLHF in Germany over a 9-year period, from 2014 to 2022. By leveraging data from the Federal Statistical Office of Germany (DESTATIS), we examine trends in morbidity, mortality, healthcare costs, and complications associated with CLHF hospitalizations. These parameters are critical for understanding the evolving landscape of CLHF management and for identifying potential areas for intervention to improve patient outcomes. Our analysis covers a period marked by significant advancements in CLHF management, including the introduction of new pharmacological therapies, such as the clinical use of angiotensin receptor-neprilysin inhibitors (ARNI) (PARADIGM-HF) and sodium-glucose cotransporter-2 inhibitors (SGLT-2i) (DAPA-HF, EMPEROR-Reduced) [3–5]. However, it is unclear how these advances have been translated into clinical practice and whether they have impacted patient outcomes on the population level. By investigating temporal trends and identifying patterns in the management and outcomes of CLHF, this study seeks to inform future healthcare policies and clinical strategies aimed at mitigating the burden of CLHF in Germany. The insights gained from this analysis will contribute to a deeper understanding of CLHF as a public health issue in Germany and guide efforts to enhance the quality of care for this vulnerable patient population.

## Methods

### Data source and study population

In Germany, federal law mandates that all hospitals submit data on in-patient hospitalizations to the Institute for the Hospital Remuneration System (Institut für das Entgeltsystem im Krankenhaus, InEK; Siegburg, Germany; http://www.g-drg.de) since 2002. A significant portion of this data has been provided for scientific research by the Federal Statistical Office. We acquired aggregated data from the Research Data Centers of the Federal Statistical Office and the Statistical Offices of the Laender (Statistisches Bundesamt, DESTATIS; https://www.destatis.de; RDC of the Federal Statistical Office and Statistical Offices of the Federal States, DOI: [10.21242/23141.2014.00.00.6.1.0 to 10.21242/23141.2022.00.00.6.1.0], own calculations) covering the years 2014 to 2022 (graphical abstract).

The dataset includes all inpatient treatments in German hospitals, organized by individual cases, with the exclusion of outpatient care or admissions to psychiatric or psychosomatic unit treatments. For every inpatient episode, the documentation of a principal diagnosis—representing the primary reason for admission—is obligatory. Additionally, any number of secondary diagnoses may be recorded to reflect the case's clinical complexity. Coding follows the International Classification of Diseases, 10th Revision, German Modification (ICD-10-GM) and the Operationen- und Prozedurenschlüssel (OPS) codes, which are maintained and updated annually by the German Institute of Medical Documentation and Information (DIMDI). All data were hosted on the secure servers of the Federal Statistical Office of Germany (DESTATIS) and were subject to strict data protection regulations. Direct access to the original anonymized dataset was not permitted. Instead, DESTATIS facilitated access by executing pre-approved analysis scripts on our behalf. To develop and validate these scripts, we were provided with a small artificial dataset replicating the structure of the original data. The finalized scripts were then submitted to DESTATIS, who executed them on the full anonymized dataset within their secure computing environment.

As documentation and transmission of these data to DESTATIS are legally mandated for reimbursement purposes within the German healthcare system, the data set can be considered comprehensive. Further information has been detailed in previous publications [6, 7]. Our dataset encompassed records of 2,616,462 cases with primary diagnoses of CLHF (ICD-10-GM I50.11-I50.14) occurring between January 1st, 2014, and December 31st, 2022. Comorbidities and procedures were defined using ICD or OPS codes. If no relevant code was identified within the hospital stay, the variable was assigned to a value of zero. Baseline characteristics for each individual were obtained (Tables 1–2), encompassing sex, classification to New York Heart Association (NYHA), cardiovascular risk factors (CVRF) (e.g., AHT, DM, dyslipidemia, obesity, nicotine abuses); cardiovascular comorbidities (e.g., coronary heart disease (CHD), peripheral artery disease (PAD), chronic kidney disease (CKD), atrial flutter (AFlu), secondary diagnosis of chronic left/right ventricular heart failure (CL/RHF), cerebrovascular disease (CeVD), previous myocardial infarction (MI), previous percutaneous coronary intervention (PCI)). Comorbidities-associated interventions recorded left-heart catheterization (LCH) and percutaneous coronary intervention (PCI). Complications were defined as cardiogenic shock, acute kidney failure (AKI), renal replacement therapy, the combination of resuscitation and left ventricular (LV) support, implantation of an assist device (extracorporeal life support, ECLS, impella, intra-aortic balloon pump (IABP)) and ventilation. Outcomes included the implantation of a uni- or biventricular intracorporeal pump, an artificial heart (total heart replacement), orthotopic or heterotopic (assist heart) heart transplantation, heart–lung-Transplantation and death. The ICD-10-GM was utilized for analyses concerning diagnoses (Online Resource 1). The OPS codes for encoding medical procedures, surgeries, and treatments (Online Resource 2).

**Table 1:** Trends in CLHF hospitalizations by year, NYHA stages, and male sex (2014–2022)

	NYHA I	NYHA II	NYHA III	NYHA IV	Total	Male
	% (*N*)	% (*N*)	% (*N*)	% (*N*)	% (*N*)	% (*N*)
2014	0.95% (2,732)	6.39% (18,412)	37.76% (108,751)	54.9% (158,124)	100% (288,019)	49.47% (142,495)
2015	0.99% (2,889)	6.18% (18,035)	37.99% (110,828)	54.84% (159,998)	100% (291,753)	49.98% (145,813)
2016	1.13% (3,331)	6.32% (18,709)	39.51% (116,922)	53.04% (156,947)	100% (295,909)	50.01% (147,989)
2017	1.09% (3,277)	6.43% (19,337)	40.29% (121,070)	52.19% (156,817)	100% (300,501)	50.07% (150,447)
2018	1.03% (2,999)	6.58% (19,159)	40,81% (118,806)	51.57% (150,127)	100% (291,091)	50.18% (146,077)
2019	1.08% (3,368)	6.82% (21,270)	41.47% (130,130)	50.36% (157,014)	100% (311,782)	50.26% (156,710)
2020	0.91% (2,479)	6.35% (17,360)	41,45% (113,289)	51.29% (140,192)	100% (273,320)	51.16% (139,824)
2021	0.85% (2,368)	6.34% (17,675)	41,5 (115,636)	51.31% (142,991)	100% (278,670)	50.72% (141,351)
2022	0.79% (2,255)	6.29% (17,958)	42,56% (121,480)	50.36% (143,724)	100% (285,417)	50.79% (144,967)
Total	0.98 (25,698)	6.42% (167,918)	40,39% (1,056,912)	52.21%( 1,365,934)	100% (2,616,462)	50.28% (1,315,673)

Absolute and relative numbers of patients hospitalized for CLHF during the observation period from 2014 to 2022, differentiated by year, NYHA stages, and male sex

*CLHF* Chronic left ventricular heart failure, *NYHA* New York heart Association

**Table 2:** Baseline cardiovascular risk factors, comorbidities, and associated interventions in CLHF patients (2014–2022) Baseline cardiovascular risk factors, comorbidities, and comorbidity-associated interventions recorded, in absolute and relative numbers, in patients hospitalised for CLHF during the observation period from 2014 to 2022, differentiated by year

	2014	2015	2016	2017	2018	2019	2020	2021	2022	*p*-value
	% (*N*)	% (*N*)	% (*N*)	% (*N*)	% (*N*)	% (*N*)	% (*N*)	% (*N*)	% (*N*)	
AHT	74.39% (214,262)	75.1% (219,107)	76.22% (225,541)	76.23% (229,162)	76.5% (222,680)	76.85% (239,615)	77.19% (210,977)	76,72% (213,806)	75.77% (216,268)	< 0.0001
Obesity	11.68% (33,655)	11.58% (33,785)	12.07% (35,712)	11.57% (34,766)	11.38% (33,124)	11.2% (34,923)	10.81% (29,557)	10.38% (28,937)	8.44% (24,090)	< 0.0001
DM	39.66% (114,240)	39.29% (114,626)	38.79% (114,780)	38.18% (114,719)	37.72% (109,799)	37.41% (116,636)	38.05% (88,547)	37.66% (104,959)	36.98% (105,538)	< 0.0001
Dyslipidemia	26.9% (77,485)	27.46% (80,112)	28.92% (85,591)	29.29% (88,007)	30.12% (87,678)	30.87% (96,240)	32.4% (88,547)	33.05% (92,096)	32.88% (93,833)	< 0.0001
Nicotine abuses	1.87% (5,379)	1.9% (5,552)	2.07% (6,137)	2.05% (6,173)	2.09% (6,076)	2.05% (6,383)	2.14% (5,855)	2.09% (5,823)	1.91% (5,440)	< 0.0001
CHD	30.45% (87,701)	31.22% (91,071)	32.68% (95,814)	32.63% (98,049)	33.11% (96,377)	33.42% (104,200)	34.31% (93,782)	34.14% (95,131)	32.77% (93,523)	< 0.0001
PAD	6.69% (19,264)	5.89% (17,177)	5.77% (17,085)	5.79% (17,397)	5.73% (16,673)	5.61% (17,479)	5.57% (15,221)	5.41% (15,069)	5.00% (14,270)	< 0.0001
CKD	47.63% (137,179)	47.73% (139,259)	48.65% (143,945)	49.09% (147,510)	49.08% (142,862)	49.29% (153,664)	50.3% (137,478)	48.88% (136,224)	46.74% (133,416)	< 0.0001
AF/AFlu	51.62% (148,672)	47.28% (153,799)	53.52% (158,384)	54.56% (163,966)	55.26% (160,843)	55.81% (173,993)	56.42% (154,210)	59.99% (158,824)	57.04% (162,801)	< 0.0001
CRHF	32.47% (92,881)	33.01% (96,304)	33.73% (99,798)	33.9% (101,882)	37.7% (101,023)	35.68% (111,248)	37.63% (102,842)	37.69% (105,023)	36.47% (104,102)	< 0.0001
CLHF	1.46% (4,203)	1.46% (4,274)	1.42% (4,197)	1.4% (4,220)	1.4% (4,062)	1.52% (4,741)	1.41% (3,841)	1.44% (4,011)	1.3% (3,699)	< 0.0001
Previous MI	8.78% (25,282)	8.92% (26,019)	9.35% (27,658)	9.39% (28,231)	9.3% (27,058)	9.07% (28,269)	8.9% (24,328)	8.48% (23,628)	7.97% (22,736)	< 0.0001
Previous PCI	17.55% (50,559)	17.9% (52,226)	18.33% (54,252)	18.08% (54,341)	18.07% (52,606)	17.97% (56,033)	18.13% (49,561)	17.58% (48,977)	16.88% (48,179)	< 0.0001
LCH	16.83% (48,468)	17.23% (50,280)	17.57% (51,995)	17.44% (52,401)	17.9% (52,100)	17.93% (55,892)	18.15% (49,595)	19.12% (53,272)	18.19% (51,888)	< 0.0001
PCI	3.6% (10,358)	3.7% (10,806)	3.92% (11,597)	3.87% (11,621)	4.01% (11,660)	4.07% (12,696)	4.34% (11,863)	4.54% (12,647)	4.22% (12,050)	< 0.0001

*AF* atrial fibrillation, *Aflu* atrial flutter, *AHT* Arterial hypertension, *CHD* coronary heart disease, *CKD* chronic kidney disease, *DM* diabetes mellitus, *LCH* left-heart catheterisation, *CL/RHF*: chronic left/right ventricular heart failure, *MI* myocardial infarction (MI), *PCI* percutaneous coronary intervention, *PAD* peripheral artery disease

**Fig. 1:**
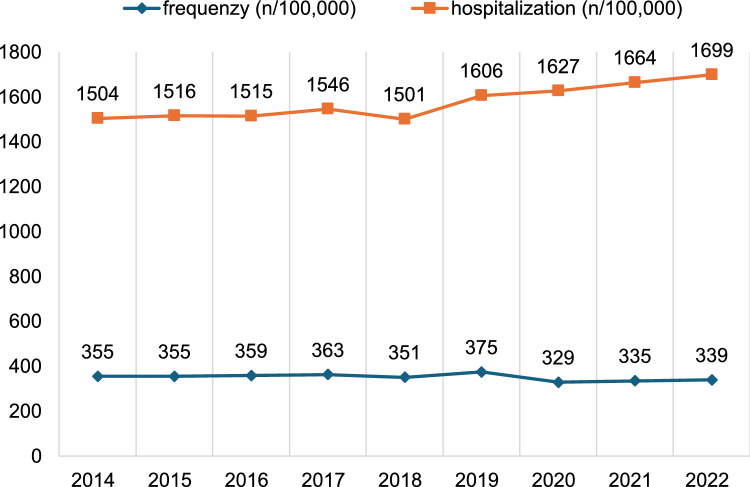
Hospitalizations due to CLHF per year in relation to population size and total hospitalizations (2014–2022). Hospitalizations due to CLHF per year are presented as hospitalizations per population (in n/100,000, presented in blue), and as n/100,000 total hospitalizations (presented in orange), irrespective of the reason for hospitalization, for the years 2014–2022. *CLHF* Chronic left ventricular heart failure

**Fig. 2:**
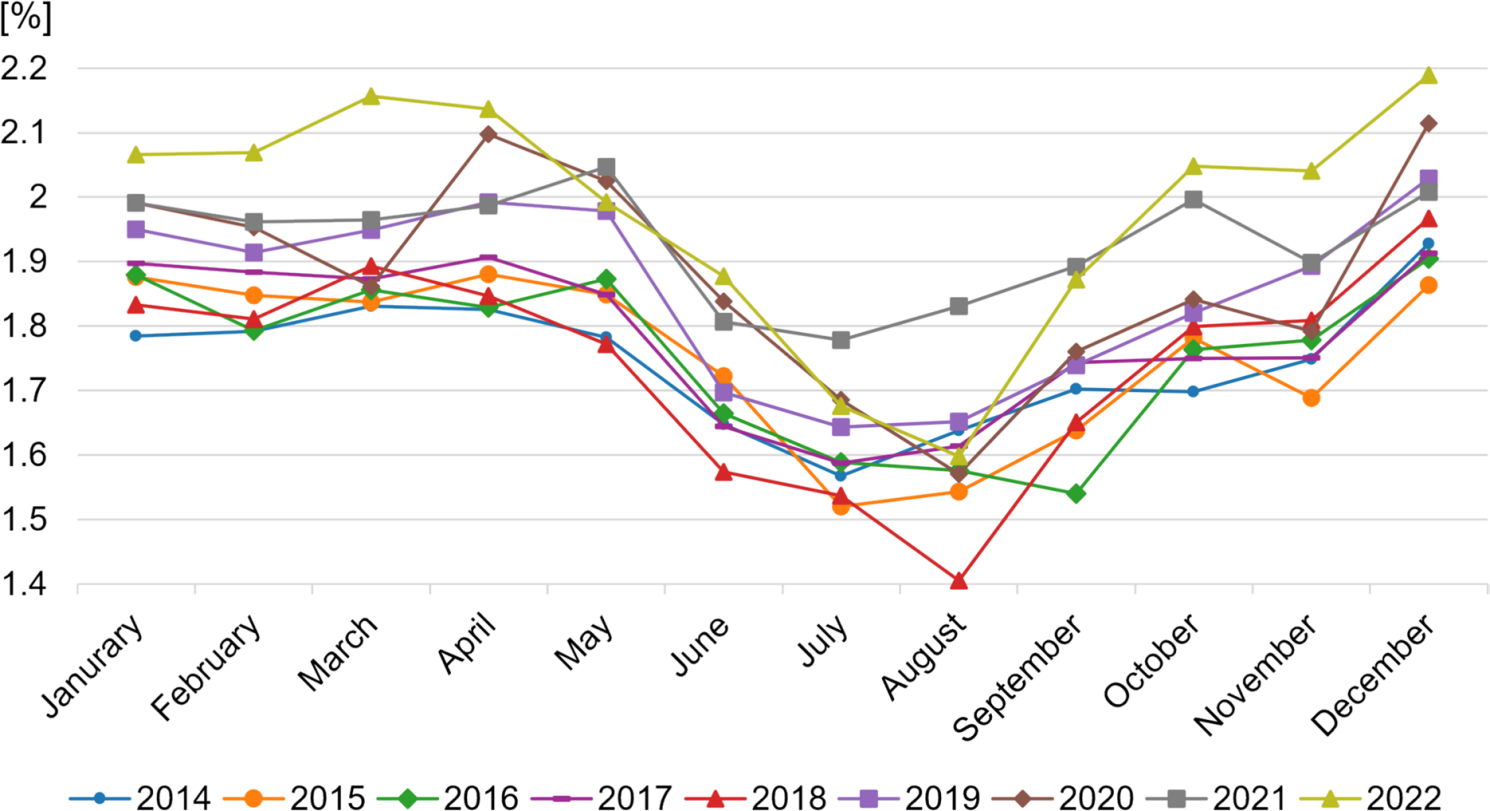
Proportion of CLHF hospitalizations per month in relation to all-caused hospitalizations (2014–2022). *CLHF* chronic left ventricular heart failure

In our analysis of hospitalization rates, we examined CLHF-related hospitalizations in relation to both the population size and the total number of hospitalizations (Fig. 1, Online Resource 3) in the respective observation years. We also analyzed the proportion of CLHF-related hospitalizations compared to cancer-related hospitalizations over the study period (Online Resource 4). Further breakdowns by age group (Online Resource 5), month (Fig. 2), and federal state (Online Resource 6) can be found in the supplementary material.

The median revenue volume, as well as the median hospitalization duration and the median age at hospitalization, were stratified by NYHA stage for the different years (Table 3).

**Table 3:** Median revenue volume, length of hospitalization, and age in CLHF patients (2014–2022)

NYHA		2014	2015	2016	2017	2018	2019	2020	2021	2022
NYHA I	revenue volume length of hospitalization age	2758.86 4.00 72.00	2804.72 4.00 73.00	2901.20 4.00 73.00	2968.11 4.00 73.00	2923.11 4.00 73.00	3013.37 4.00 74.00	2347.96 4.00 74.00	2454.26 4.00 74.00	2497.91 4.00 74.00
NYHA II	revenue volume length of hospitalization age	2758.86 4.00 75.00	2804.72 4.00 75.00	2901.20 4.00 75.00	2968.10 4.00 76.00	2923.53 4.00 77.00	3013.22 4.00 77.00	2348.07 4.00 77.00	2456.35 4.00 78.00	2497.91 4.00 78.00
NYHA III	revenue volume length of hospitalization age	2781.71 7.00 78.00	2804.72 7.00 79.00	2901.20 7.00 79.000	2968.91 7.00 79.00	2931.34 7.00 80.00	3017.06 7.00 80.00	2348.98 7.00 80.00	2457.32 7.00 81.00	2498.78 7.00 81.00
NYHA IV	revenue volume length of hospitalization age	2821.38 9.00 80.00	2836.22 9.00 80.00	2901.20 9.00 80.00	2972.53 9.00 81.00	2931.34 8.00 81.00	3017.06 8.00 81.00	2350.05 8.00 81.00	2459.61 8.00 82.00	2499.54 8.00 82.00

Median revenue volume in euro, median length of hospitalization due to CLHF in days, and the median age in years, differentiated by NYHA stage and year of observation

*NYHA* New York heart Association

### Statistics

Our analysis included all inpatient CLHF cases in Germany from 2014 to 2022 and does not represent a subset of data. Regarding our primary research question, we aimed to examine time trends during the observation period of 2014–2022. Therefore, time-trends for categorical variables were tested using the Mantel–Haenszel Chi-square test and time-trends for continuous variables were analyzed using Spearman correlation analysis. Categorical variables between groups (e.g. between NYHA stages) were tested via a two-sided Chi-square test, whereas continuous variables were contrasted between groups using the two-sided Wilcoxon test. All analyses conducted were fully exploratory and all p-values were interpreted accordingly. Specifically, all p-values were two-sided and p-values less than 0.05 were considered statistically noticeable. The statistical analysis was executed using SAS software (SAS 9.3: SAS Institute Inc., Cary, NC, USA), and R version 4.0.2 (2020-06-22).

## Results

We included a total of 2,616,462 cases hospitalized with CLHF in our study (graphical abstract). Over time, the absolute number of CLHF cases increased steadily from 2014 to 2019 (2014: 288,019; 2019: 311,782; + 8.25%), with a slight dip in 2018 (291,091), before a decline in hospitalizations occurred in 2020 (273,320) (Table 1). A similar pattern is evident when considering CLHF hospitalization numbers in relation to cancer-related hospitalizations (Online Resource 4). From 2021 onwards, the absolute case numbers began to rise again but remained below the 2019 peak. A similar trend was observed for the number of CLHF cases relative to the total German population (Fig. 1, Online Resource 3). However, when the CLHF hospitalization numbers are considered in relation to the total hospitalizations of the corresponding year, the observed decline in CLHF hospitalizations in 2020 is not evident. CLHF-related hospitalizations per 100,000 total hospitalizations show a continuous upward trend until 2022, with the exception of a slight dip in 2018 (Fig. 1, Online Resource 3).

A more detailed breakdown of the proportion of CLHF hospitalizations relative to all-cause and cancer-related hospitalizations across different age groups is provided in Online Resource 5. Overall, the proportion of CLHF hospitalizations compared to cancer-related hospitalizations increases consistently with advancing age across all years. Within each age group, a general upward trend in CLHF hospitalizations can also be observed over time, with a transient decline occurring between 2016 and 2018 (varying slightly by age group) and again in 2020.

When examining the proportion of hospitalizations due to CLHF relative to all-cause hospitalizations over the course of the year, differentiated by month, a seasonal pattern becomes apparent. Depending on the year of observation, a decline in hospitalizations typically begins around May or June, followed by a renewed increase between August and October. With the exception of 2015 (March) and 2021 (May), the proportion of CLHF hospitalizations was highest in December and, depending on the year, lowest between July and September (Fig. 2).

**Fig. 3:**
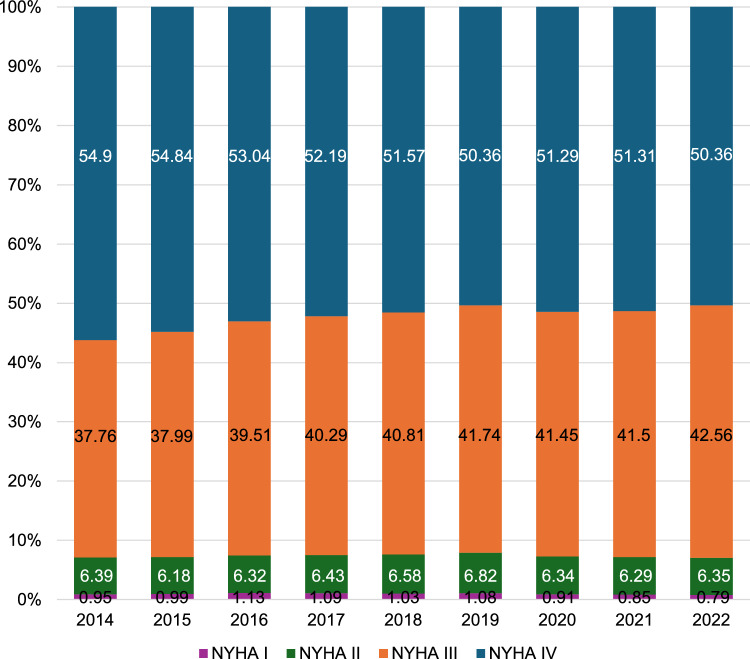
Proportions of NYHA stages in hospitalizations due to CLHF (2014–2022). Representation of the percentage distribution of the different NYHA stages in patients hospitalized due to chronic left ventricular heart failure over the observation period from 2014 to 2022 in percentage. *CLHF* Chronic left ventricular heart failure, NYHA: New York Heart Association

The gender distribution was overall balanced (male: 50.29% ± 0.9% (1,315,700) vs. female: 49.71% ± 0.9% (1,300,762)) and remained relatively constant over time (Table 1). The lowest proportion of male patients was recorded in 2014 at 49.02%, while the highest was in 2020 at 51.16% (Table 1). Our data predominantly represent patients with advanced CLHF; more than half (52.21%; *N* = 1,365,934) were classified as NYHA stage IV, while less than one percent (0.98%; *N* = 25,698) were asymptomatic (Table 1, Fig. 3). Nevertheless, over time, a decline in the proportion of highly symptomatic patients was documented, from 54.90% (158,124) classified as NYHA IV in 2014 to 50.36% (143,724) in 2022 (*p* < 0.0001) (Table 1, Fig. 3).

### Trends in concomitant diseases

Over time, AHT, obesity, DM, CKD, and AF/AFlu were common comorbidities. AHT was coded as a comorbidity in approximately three-quarters of all patients, peaking at 77.19% in 2020 (Table 2). Notably, the prevalence of atrial AF/AFlu increased among those hospitalized for CLHF, rising from 51.62% (148,672) in 2014 to 57.04% (162,801) in 2022 (*p* < 0.0001) (Table 2). Interestingly, the proportion of overweight patients hospitalized for CLHF decreased over time (*p* < 0.0001), with a maximum of 12.07% (35,712) in 2016 and a minimum of 8.44% (24,090) in 2022 (Table 2). Similarly, the diagnosis of DM declined, from a maximum of 39.66% in 2014 to a minimum of 36.98% in 2022 (*p* < 0.0001) (Table 2). The prevalence of CKD steadily increased until 2020 (47.63% in 2014; 50.3% in 2020; *p* < 0.0001), before subsequently declining to 46.74% (133,416) in 2022 (Table 2). The comorbidity with CHD increased steadily from 2014 (30.45%) to 2020 (34.31%; *p* < 0.0001) before slightly declining again in 2022 (32.77%; *p* < 0.0001)) (Table 2). Alongside this trend, the percentage of in-hospital CLHF patients who underwent cardiac catheterization and coronary interventions also increased (Table 2). Although most CLHF cases worldwide are of ischemic etiology, the patient data presented does not allow us to draw conclusions about the relationship between CHD and coronary interventions in the context of ischemic cardiomyopathies. The data source lacks specific coding for CLHF etiology, limiting our ability to make further conclusions.

### Trends in in-hospital complications and in in-hospital mortality

Over the years, there was an increase in the incidence of cardiogenic shock among patients hospitalized with CLHF (2014: 1.02%; 2020: 1.31%; *p* < 0.0001) (Online Resource 7, Fig. 4g). Similarly, the 2020s saw a rise in the use of resuscitation and implanted left ventricular support systems (minimum 1.32% in 2014; maximum 1.49% in 2020; *p* = 0.003) (Online Resource 7, Fig. 4h). The percentage of patients receiving assist devices (ECLS, Impella, IABP) more than doubled during the observation period (*p* < 0.0001) (Online Resource 7, Fig. 4d). The proportion of patients requiring mechanical ventilation remained consistent over the years at just over 6 ± 0.3% (Online Resource 7, Fig. 4b). Univentricular heart support systems were rarely implanted, ranging from 0.08% in 2015 to 0.12% in 2019, while the rate of orthotopic heart transplants remained between 0% and 0.02% across all years (Online Resource 7, Fig. 4e,f). The percentage of patients with documented AKI more than doubled (2014: 6.47%; 2021: 13.03%; p < 0.0001), accompanied by a trend of increasing dialysis dependence (*p* < 0.0001) (Online Resource 7, Fig. 4a). Overall, the total in-hospital mortality rate was 8.12%. From 2015 to 2019, in-hospital mortality rate decreased (although the absolute annual number of in-hospital deaths remained consistently high) from 8.03% (23,137) in 2014 to 7.61% in 2019 (23,719) (*p* < 0.0001) (Online Resource 7, Fig. 4C). From 2020 onwards, there was a resurgence in the in-hospital mortality rate with a maximum of 8.84% (25,220) (*p* < 0.0001) in 2022 (Online Resource 7, Fig. 4c).

**Fig. 4:**
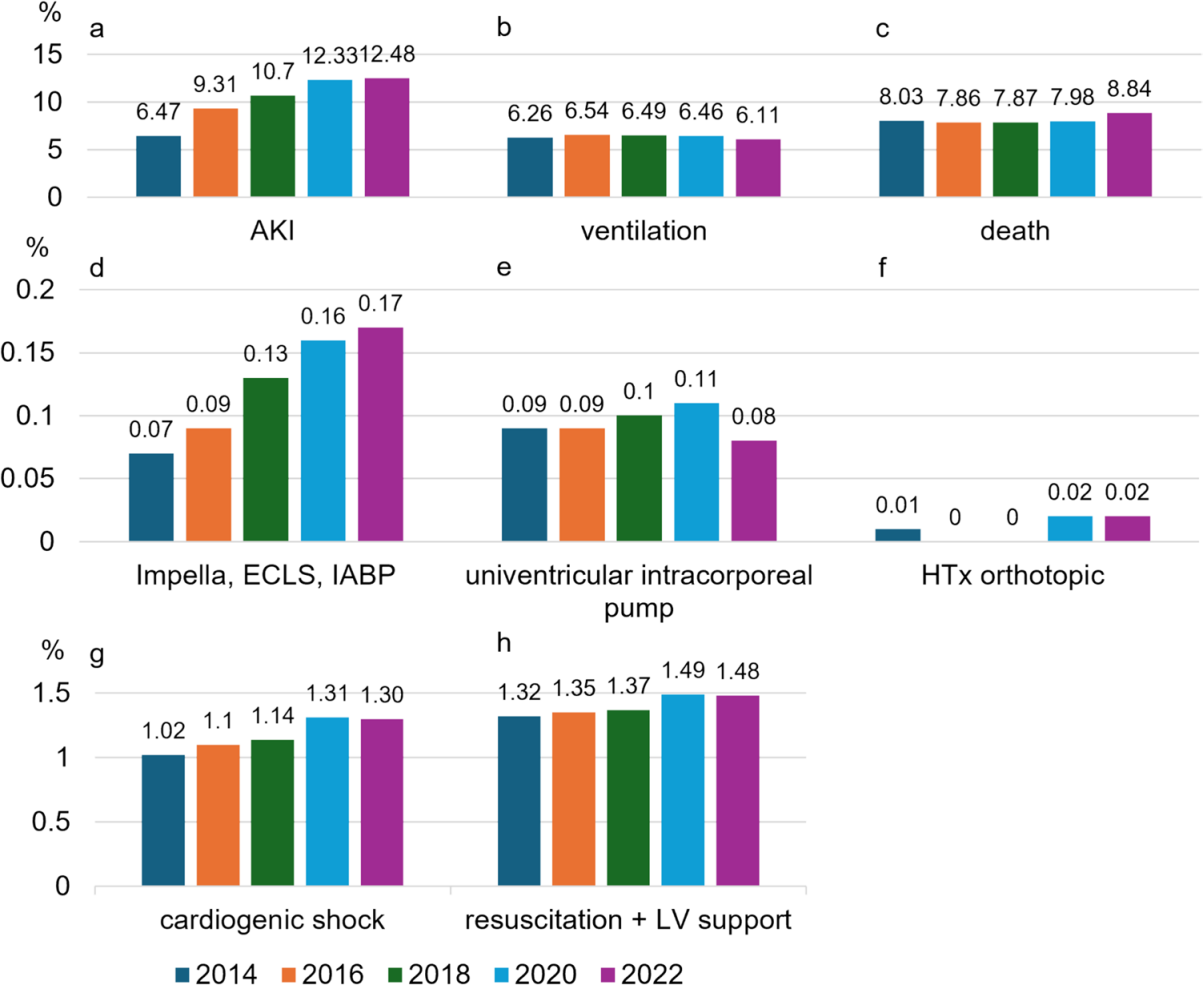
Complication in hospitalized CLHF patients (2014–2022). Complications in patients during hospitalization for CLHF over the observation period from 2014 to 2022, presented in relative numbers, differentiated by year. *AKI* acute kidney failure, *CLHF* Chronic left ventricular heart failure, *HTx* heart transplantation, *IABP* intra-aortic balloon pump, *LV* left ventricular, *ECLS* extracorporeal life support (ECLS)

### Trends in median revenue volume and hospitalization duration

Our cohort data indicate that the revenue volume, and thus the costs for health insurance providers increased across all NYHA stages from 2014 to 2020 (*p* < 0.0001) (Table 3). However, in 2020, there was a marked decline in the median revenue volume, dropping below the 2014 baseline, possibly due to changes in the patient population brought about by the COVID-19 pandemic, including a shift in the median revenue volume towards more complex treatments and the impact of specific financial support measures during this period (Table 3). From 2021 onwards, the median revenue volume increased again across all NYHA stages but remained below the peak values observed in 2019. In all years, patients in higher NYHA stages were, in median older than in lower ones (*p* < 0.0001) (Table 3). Across all NYHA stages, the median age of in-hospital CLHF patients at the time of hospitalization increased over the observation period (*p* < 0.0001) (Table 3). The median length of hospitalization due to CLHF varied according to the NYHA stage in all observation years, with longer stays observed in higher NYHA stages compared to lower ones (Δ max: + 5 days in NYHA I vs NYHA IV from 2014 to 2017; *p* < 0.0001) (Table 3). The median length of hospitalization remained stable across the NYHA stages over time, with a maximum variation of 1 day, in median.

## Discussion

This study provides a comprehensive analysis of temporary in-hospital outcomes for CLHF patients in Germany from 2014 to 2022, revealing critical trends in morbidity, mortality, healthcare costs, and complications. These findings contribute to a deeper understanding of the evolving burden of CLHF, the effects of recent therapeutic advances, and our current standing in the management of CLHF.

Our data reveal a steady increase in CLHF hospitalizations from 2014 to 2019. Although a comparison of temporal trends in CLHF-related hospitalizations across different countries is difficult due to the varying standards of different nations, the ongoing increase in CLHF was also observed in the United States [8, 9]. Compared to other countries, there is little data available on the trends in hospitalization of CLHF patients after 2014. It is unclear whether the observed increase is due to a genuine increase in CLHF hospitalizations, changes in coding practices, improved diagnostic processes, or growing awareness of CLHF with preserved ejection fraction. In the years before 2014, the data about CLHF hospitalization varied by country, ranging from decreasing to constant to increasing [10–13]. In Germany, the trend of increasing hospitalizations due to CLHF was already observed from 2000 to 2013 [14].

Our observed increase of CLHF-hospitalization from 2014 to 2019 was followed by a significant decline of CLHF-hospitalizations in 2020 and again a rise thereafter. This decline aligns with global trends observed during the COVID-19 pandemic, where healthcare systems faced unprecedented challenges, including reduced hospital admissions for chronic conditions like CLHF [15, 16]. Studies have shown that many patients avoided seeking hospital care during the pandemic due to fears of infection and restrictions on elective procedures, which likely contributed to the reduction in absolute CLHF hospitalizations [17, 18]. This reduction may have led to delayed treatments and exacerbations of CLHF in patients, potentially influencing the observed increase in in-hospital mortality from 2020 onwards. However, the relative share of CLHF-related hospitalizations compared to overall and cancer-related hospital admissions increased, indicating that CLHF remained a significant contributor to inpatient care.

Furthermore, when examining the proportion of CLHF hospitalizations relative to all-cause hospital admissions throughout the year, a consistent seasonal pattern emerged. Across most years, a decline typically began in May or June, followed by an increase between August and October. Notably, with the exception of 2015 (March) and 2021 (May), the highest monthly proportion of CLHF hospitalizations was observed in December, while the lowest values were generally recorded between July and September, depending on the year. These seasonal fluctuations may reflect environmental influences, such as colder temperatures and increased respiratory infections in winter, as well as behavioral factors and hospital system dynamics during the summer months.

The absolute number of CLHF deaths throughout the entire observation period ranged between 21,819 and 25,220 per year. Nevertheless, our data show a decrease in mortality rates from 2014 to 2019, likely reflecting changes in CLHF therapies. The potential impact of the increasing use of mechanical circulatory support devices, such as ECLS and ventricular assist devices, on in-hospital mortality warrants further discussion. The growing reliance on these advanced technologies underscores their critical role in managing acute CLHF episodes, yet their influence on patient outcomes, including mortality, remains an important consideration. However, these interventions are often associated with significant risks and resource use, underscoring the need for careful patient selection and optimization of care pathways.

Although our data predominantly reflects patients with advanced CLHF, with over half of the cohort (52.21%) classified as NYHA stage IV, it is noteworthy that there was a gradual decline in the proportion of highly symptomatic patients over time, decreasing from 54.90% in 2014 to 50.36% in 2020. Based on the available data, it is unclear whether this trend should be seen as progress, with CLHF patients being hospitalized at earlier NYHA stages—enabling the earlier detection of complications or impending poor outcomes—or as a setback, where the increasing comorbidities and aging CLHF population are making hospitalization necessary at earlier NYHA stages. However, the persistence of a substantial proportion of patients in NYHA stage IV and the increasing number of CLHF-hospitalizations in total underscores the ongoing challenges in managing advanced CLHF and highlights the need for continued efforts to improve outcomes for this vulnerable patient population. Further, the subsequent rise in mortality rates during the COVID-19 pandemic underlines the vulnerability of CLHF patients during periods of healthcare system strain and highlights the need for targeted strategies to ensure continuous care for chronic conditions during such crises.

The trends in comorbidities among CLHF patients are also notable. The increasing prevalence of AF/AFlu and CKD observed in our cohort mirrors global patterns and emphasizes the complex interplay of CLHF with other chronic conditions [1]. The decline in the prevalence of diabetes and obesity among hospitalized CLHF patients may reflect shifting patient demographics or changes in diagnostic coding practices, though these trends warrant further investigation to understand their implications fully. Complication rates, including cardiogenic shock and AKI, have risen over the observation period, particularly in 2020. The increase in CLHF-related cardiogenic shock is particularly concerning given its association with high mortality and significant healthcare costs [19]. Similarly, the increase in AKI and dialysis dependency is alarming, as both are associated with higher morbidity risks and substantial cost implications. Studies have already noted a rise in AKI and renal replacement therapy in the years preceding our observation period [20]. The increase in these complications underscores the multimorbidity and complexity of patients hospitalized with CLHF.

The rise in CLHF-associated complications and the increased use of mechanical circulatory support devices go hand-in-hand with a corresponding increase in hospitalization costs. The observed trends in the median revenue volume and hospitalization duration reflect broader shifts in healthcare economics and resource allocation. The increase in revenue volume across all NYHA stages until 2020 suggests rising costs for managing CLHF, consistent with the growing complexity of CLHF care. The subsequent decline in revenue volume in 2020 may be attributed to changes in patient median revenue volume due to the pandemic, including fewer elective admissions and more urgent, complex cases, as well as the financial impact of COVID-19-related healthcare policies.

In summary, this study highlights the persistent challenges in managing CLHF despite therapeutic advancements. The data reveal rising hospitalization rates, complications, and healthcare costs, particularly up to 2019, with a notable impact from the COVID-19 pandemic. While newer therapies have reduced mortality rates, the ongoing complexity of CLHF, including increased comorbidities and reliance on advanced support devices, underscores the need for continued innovation and optimized care strategies. These findings provide a crucial foundation for improving CLHF outcomes in the future.

### Limitations

Regarding the interpretability of the data presented, several limitations should be acknowledged. First, the data are specific to Germany, making them most relevant within this national context and potentially applicable only to countries with similar healthcare systems. The analysis is confined to in-hospital data, lacking information on outpatient care and post-discharge outcomes, which limits the understanding of comprehensive CLHF management and patient recovery. Furthermore, the hospitalization statistics are case-based rather than patient-based, meaning that the count reflects all cases within a year and does not allow for definitive conclusions about the number of unique patients affected, though this does not impact the accuracy of the absolute number of in-hospital deaths. Additional limitations include potential coding errors and changes in coding practices over time, especially concerning CLHF with preserved ejection fraction. The impact of external factors, such as changes in treatment guidelines and public health interventions, may also influence the observed trends. Lastly, about other important outcomes such as quality of life and readmission rates are no data available, and unmeasured variables, such as socioeconomic status, may introduce bias. These limitations should be considered when interpreting the study’s findings and applying them to broader contexts.

## Supplementary Information

Below is the link to the electronic supplementary material.

Supplementary file1 (DOCX 395 KB)

## References

[1] Ziaeian B, Fonarow GC (2016) Epidemiology and aetiology of heart failure. Nat Rev Cardiol 13(6):368–378. 10.1038/nrcardio.2016.25 26935038 10.1038/nrcardio.2016.25 PMC4868779

[2] Schelhase T (2024) Statistische Krankenhausdaten: Diagnosedaten der Krankenhauspatienten. In: Klauber J, Wasem J, Beivers A, Mostert C, Scheller-Kreinsen D (eds) Krankenhaus-Report. Springer, Berlin, Heidelberg, pp 465–497

[3] McMurray JJ, Packer M, Desai AS, Gong J, Lefkowitz MP, Rizkala AR, PARADIGM-HF Investigators and Committees et al (2014) Angiotensin-neprilysin inhibition versus enalapril in heart failure. N Engl J Med 371(11):993–1004. 10.1056/NEJMoa1409077 25176015 10.1056/NEJMoa1409077

[4] McMurray JJV, Solomon SD, Inzucchi SE, Køber L, Kosiborod MN, Martinez FA et al (2019) Dapagliflozin in patients with heart failure and reduced ejection fraction. N Engl J Med 381(21):1995–2008. 10.1056/NEJMoa1911303 31535829 10.1056/NEJMoa1911303

[5] Packer M, Anker SD, Butler J, Filippatos G, Pocock SJ, Carson P et al (2020) Cardiovascular and renal outcomes with empagliflozin in heart failure. N Engl J Med 383(15):1413–1424. 10.1056/NEJMoa2022190 32865377 10.1056/NEJMoa2022190

[6] Freisinger E, Fuerstenberg T, Malyar NM, Wellmann J, Keil U, Breithardt G et al (2014) German nationwide data on current trends and management of acute myocardial infarction: discrepancies between trials and real-life. Eur Heart J 35(15):979–988. 10.1093/eurheartj/ehu043 24558113 10.1093/eurheartj/ehu043

[7] Kuehnemund L, Koeppe J, Feld J, Wiederhold A, Illner J, Makowski L et al (2021) Gender differences in acute myocardial infarction-A nationwide German real-life analysis from 2014 to 2017. Clin Cardiol 44(7):890–898. 10.1002/clc.23662 34075604 10.1002/clc.23662 PMC8259152

[8] Agarwal MA, Fonarow GC, Ziaeian B (2021) National trends in heart failure hospitalizations and readmissions from 2010 to 2017. JAMA Cardiol 6(8):952–956. 10.1001/jamacardio.2020.7472 33566058 10.1001/jamacardio.2020.7472 PMC7876620

[9] Clark KAA, Reinhardt SW, Chouairi F, Miller PE, Kay B, Fuery M et al (2022) Trends in heart failure hospitalizations in the US from 2008 to 2018. J Card Fail 28(2):171–180. 10.1016/j.cardfail.2021.08.020 34534665 10.1016/j.cardfail.2021.08.020

[10] Brettell R, Soljak M, Cecil E, Cowie MR, Tuppin P, Majeed A (2013) Reducing heart failure admission rates in England 2004–2011 are not related to changes in primary care quality: national observational study. Eur J Heart Fail 15:1335–1342. 10.1093/eurjhf/hft107 23845798 10.1093/eurjhf/hft107 PMC3834843

[11] Wasywich CA, Gamble GD, Whalley GA, Doughty RN (2010) Understanding changing patterns of survival and hospitalization for heart failure over two decades in New Zealand: utility of ‘days alive and out of hospital’ from epidemiological data. Eur J Heart Fail 12:462–468. 10.1093/eurjhf/hfq027 20194215 10.1093/eurjhf/hfq027

[12] Paren P, Schaufelberger M, Bjorck L, Lappas G, Fu M, Rosengren A (2014) Trends in prevalence from 1990 to 2007 of patients hospitalized with heart failure in Sweden. Eur J Heart Fail 16:737–742. 10.1002/ejhf.109 24863749 10.1002/ejhf.109

[13] Gigli G, Lispi L, Donati C, Orlandi S, Vallebona A, Gigli L et al (2009) Trends in hospitalization for heart failure in Italy 2001–2003. J Cardiovasc Med 10:367–371. 10.2459/JCM.0b013e3283276e1c 10.2459/JCM.0b013e3283276e1c 19318978

[14] Christ M, Störk S, Dörr M, Heppner HJ, Müller C, Wachter R et al (2016) Trend HF Germany Project. Heart failure epidemiology 2000–2013: insights from the German federal health monitoring system. Eur J Heart Fail 18(8):1009–1018. 10.1002/ejHF.567 27246139 10.1002/ejhf.567

[15] Rosenbaum L (2020) The untold toll - the pandemic’s effects on patients without Covid-19. N Engl J Med 382(24):2368–2371. 10.1056/NEJMms2009984 32302076 10.1056/NEJMms2009984

[16] Santi L, Golinelli D, Tampieri A, Farina G, Greco M, Rosa S et al (2021) Non-COVID-19 patients in times of pandemic: emergency department visits, hospitalizations and cause-specific mortality in Northern Italy. PLoS ONE 16(3):e0248995. 10.1371/journal.pone.0248995 33750990 10.1371/journal.pone.0248995 PMC7984614

[17] Solomon MD, Nguyen-Huynh M, Leong TK, Alexander J, Rana JS, Klingman J et al (2021) (2021) Changes in patterns of hospital visits for acute myocardial infarction or ischemic stroke during COVID-19 surges. JAMA 326(1):82–84. 10.1001/jama.2021.8414 34076670 10.1001/jama.2021.8414 PMC8173470

[18] Bromage DI, Cannatà A, Rind IA, Gregorio C, Piper S, Shah AM et al (2020) The impact of COVID-19 on heart failure hospitalization and management: report from a heart failure unit in London during the peak of the pandemic. Eur J Heart Fail 22(6):978–984. 10.1002/ejHF.1925 32478951 10.1002/ejhf.1925 PMC7300902

[19] Ghajar A, Ordonez CP, Philips B, Pinzon PQ, Fleming LM, Motiwala SR et al (2022) Cardiogenic shock related cardiovascular disease mortality trends in US population: heart failure vs. acute myocardial infarction as contributing causes. Int J Cardiol 367:45–48. 10.1016/j.ijcard.2022.08.043 36002041 10.1016/j.ijcard.2022.08.043

[20] Correa A, Patel A, Chauhan K, Shah H, Saha A, Dave M et al (2018) National trends and outcomes in dialysis-requiring acute kidney injury in heart failure: 2002–2013. J Card Fail 24(7):442–450. 10.1016/j.cardfail.2018.05.001 29730235 10.1016/j.cardfail.2018.05.001

