# Supplementary material for: Evolving trends and persistent challenges in chronic left ventricular heart failure management: insights from a 9-year analysis of in-hospital outcomes in Germany
Source: Clin Res Cardiol. 2025 May 26;115(9):1522–32. doi: 10.1007/s00392-025-02679-4 (PMC13457485; doi:10.1007/s00392-025-02679-4)
Supplement: Supplementary file 1 [file 392_2025_2679_MOESM1_ESM.docx]

**Clinical Research in Cardiology**

**Evolving Trends and Persistent Challenges in Chronic Left Ventricular Heart Failure Management: Insights from a Nine-Year Analysis of In-Hospital Outcomes in Germany**

Anastasia J. Hobbach^1^, Jannik Feld^2^, Jürgen R. Sindermann^1^, Holger Reinecke^1^

^1^Department of Cardiology I, Coronary, Peripheral Vascular Disease and Heart Failure, University Hospital Münster, Münster, Germany

^2^Institute of Biostatistics and Clinical Research, University of Münster, Münster, Germany

Correspondence to Anastasia J. Hobbach

^1^Department of Cardiology I, Coronary, Peripheral Vascular Disease and Heart Failure, University Hospital Münster, Albert-Schweizer Campus 1, 48149 Münster, Germany

ORCID: 0000-0001-6421-8429

Email: Anastasia.Hobbach@ukmuenster.de

**Online Resource 1.** Used Health Problems 10^th^ Revision (ICD-10 GM) codes.

| Health Problems 10th Revision (ICD-10 GM) | Code |
| --- | --- |
| Arterial hypertension (AHT) | I10.-, I11.-, I12.-, I13.-, I15.- |
| Diabetes mellitus (DM) | E10.-, E11.-, |
| Dyslipidemia | E78.- |
| Nicotine abuses | F17.- |
| Myocardial Infarction (MI) | I25.2, I24.1 |
| Obesity | E66.- |
| Chronic right ventricular heart failure (CRHF) | I50.11- I50.14 |
| Chronic left ventricular heart failure (CLHF) | I50.1- |
| Previous stroke | I69.3, I69.4 |
| Coronary heart disease (CHD) | I25.- |
| Atrial flutter/fibrillation (AFL/AF) | I48.- |
| Peripheral artery disease (PAD) | I70.2- |
| Cerebrovascular disease (CeVD) | I65.-, |
| Chronic kidney disease (CKD) | N18.-, N19.- |
| Acute kidney injury (AKI) | N17 |
| Cardiogenic shock | R57.0, T81.1 |
| Cancer | C00–C97 |

**Online Resource 2.** Used German procedure classification system (OPS) codes.

| German procedure classification system (OPS) | Code |
| --- | --- |
| Percutaneous coronary intervention (PCI) | 8-837 |
| Left-heart-Catheterization (LCH) | 1-275 |
| Biventricular intracoporal pump | 5-376.50 \| |
| Univentricular intracoporal pump | 5-376.40 |
| Impella | 8-83a.3 |
| Artificial heart | 5-376.6 \| |
| HTx heterotopic | 5-375.1 |
| HTx orthotopic | 5-375.0 |
| Heart- and Lung Tx | 5-375.2 |
| Intra-aortic ballon pump (IABP) | 8-83a.0 |
| Renal replacement therapy | 8-853, 8-855, 8-854, 8-857 |
| Left ventricle support | 8-851, 8-852, 8-83a.0, 8-83a.3 |
| Resuscitation | 8-771 |

**Online Resource 3: Hospitalizations due to CLHF per year: absolute numbers, per 100,000 population, and total hospitalizations (2014–2022).** Hospitalizations due to CHF per year are presented as absolute numbers, as hospitalizations per population (in n/100,000), and as per 100,000 total hospitalizations, irrespective of the reason for hospitalization, for the years 2014–2022. CLHF: Chronic left ventricular heart failure.

|  | **2014** | **2015** | **2016** | **2017** | **2018** | **2019** | **2020** | **2021** | **2022** |
| --- | --- | --- | --- | --- | --- | --- | --- | --- | --- |
| **CLHF hospital admissions** | 288,019 | 291,753 | 295,909 | 300,501 | 291,091 | 311,782 | 273,320 | 278,640 | 285,417 |
| **total hospital admissions** | 19,148,626 | 19,239,574 | 19,532,779 | 19,442,810 | 19,392,466 | 19,415,555 | 16,793,962 | 16,742,344 | 16,802,693 |
| **hospitalization (n/100.000)** | 1,504 | 1,516 | 1,515 | 1,546 | 1,501 | 1,606 | 1,627 | 1,664 | 1,699 |
| **population number** | 81,197,537 | 82,175,684 | 82,521,653 | 82,792,351 | 83,019,213 | 83,166,711 | 83,155,031 | 83,237,124 | 84,358,845 |
| **frequenzy (n/100.000)** | 355 | 355 | 359 | 363 | 351 | 375 | 329 | 335 | 339 |

**Online Resource 4: Proportion of hospitalizations due to CLHF in relation to all hospitalizations and to cancer related hospitalization (2014–2022).** The proportion of hospitalizations due to CLHF in relation to all hospitalizations (presented in blue) and to cancer related hospitalization (presented in orange) for the years 2014–2022. CLHF: chronic left ventricular heart failure**.**

**Online Resource 5**: Proportion of CLHF hospitalization compared to all-caused hospitalizations and compared to cancer-related hospitalizations by various age groups in years. CLHF: Chronic left ventricular heart failure.

| Age |  |  | 2014 | 2015 | 2016 | 2017 | 2018 | 2019 | 2020 | 2021 | 2022 |
| --- | --- | --- | --- | --- | --- | --- | --- | --- | --- | --- | --- |
| <40 | CLHF:all |  | 0.05 | 0.05 | 0.05 | 0.01 | 0.06 | 0.06 | 0.06 | 0.07 | 0.07 |
|  | CLHF:cancer |  | 2.70 | 2.87 | 2.95 | 3.13 | 3.03 | 3.27 | 2.96 | 3.28 | 3.30 |
| 40-49 | CLHF:all |  | 0.26 | 0.27 | 0.29 | 0.28 | 0.29 | 0.30 | 0.32 | 0.32 | 0.33 |
|  | CLHF:cancer |  | 4.11 | 4.32 | 4.65 | 4.72 | 4.72 | 5.04 | 4.79 | 4.99 | 5.01 |
| 50-59 | CLHF:all |  | 0.62 | 0.62 | 0.62 | 0.61 | 0.61 | 0.62 | 0.63 | 0.65 | 0.67 |
|  | CLHF:cancer |  | 5.88 | 5.97 | 6.01 | 5.96 | 5.94 | 6.02 | 5.64 | 5.90 | 6.12 |
| 60-69 | CLHF:all |  | 1.27 | 1.27 | 1.29 | 1.27 | 1.21 | 1.27 | 1.27 | 1.26 | 1.23 |
|  | CLHF:cancer |  | 9.30 | 9.39 | 9.57 | 9.40 | 8.94 | 9.25 | 8.57 | 8.55 | 8.66 |
| 70-79 | CLHF:all |  | 2.40 | 2.40 | 2.40 | 2.38 | 2.28 | 2.36 | 2.36 | 2.37 | 2.31 |
|  | CLHF:cancer |  | 20.22 | 20.51 | 20.37 | 19.90 | 18.79 | 19.05 | 17.52 | 17.50 | 17.64 |
| 80-89 | CLHF:all |  | 4.37 | 4.29 | 4.26 | 4.28 | 4.11 | 4.32 | 4.28 | 4.40 | 4.39 |
|  | CLHF:cancer |  | 20.22 | 20.51 | 20.37 | 19.90 | 18.79 | 19.05 | 17.52 | 17.50 | 17.64 |
| >90 | CLHF:all |  | 5.73 | 5.62 | 5.72 | 5.79 | 5.69 | 6.17 | 6.10 | 6.56 | 6.77 |
|  | CLHF:cancer |  | 134.53 | 138.40 | 137.73 | 137.96 | 135.94 | 143.33 | 139.65 | 144.79 | 159.02 |

**Online Resource 6:** Proportion of CLHF hospitalizations per year in relation to all-caused hospitalizations differentiated by federal state (2014-2022). CLHF: chronic left ventricular heart failure.

| Federal state | 2014 | 2015 | 2016 | 2017 | 2018 | 2019 | 2020 | 2021 | 2022 |
| --- | --- | --- | --- | --- | --- | --- | --- | --- | --- |
| Schleswig-Holstein | 1.61 | 1.64 | 1.60 | 1.68 | 1.65 | 1.74 | 1.73 | 1.79 | 1.92 |
| Hamburg | 1.59 | 1.54 | 1.52 | 1.51 | 1.40 | 1.67 | 1.74 | 1.68 | 1.84 |
| Lower Saxony | 1.86 | 1.85 | 1.85 | 1.96 | 1.95 | 2.10 | 2.13 | 2.25 | 2.38 |
| Bremen | 1.26 | 1.49 | 1.50 | 1.40 | 1.43 | 1.68 | 2.04 | 2.18 | 2.35 |
| North Rhine-Westphalia | 1.66 | 1.73 | 1.73 | 1.75 | 1.73 | 1.83 | 1.83 | 1.92 | 2.00 |
| Hesse | 1.56 | 1.50 | 1.51 | 1.52 | 1.41 | 1.49 | 1.52 | 1.62 | 1.69 |
| Rhineland-Palatinate | 1.76 | 1.76 | 1.69 | 1.77 | 1.77 | 1.82 | 1.86 | 1.94 | 1.90 |
| Baden-Württemberg | 1.59 | 1.56 | 1.59 | 1.66 | 1.64 | 1.75 | 1.71 | 1.76 | 1.78 |
| Bavaria | 1.51 | 1.47 | 1.45 | 1.43 | 1.37 | 1.44 | 1.40 | 1.46 | 1.43 |
| Saarland | 1.49 | 1.41 | 1.41 | 1.39 | 1.37 | 1.44 | 1.44 | 1.51 | 1.62 |
| Berlin | 1.57 | 1.48 | 1.48 | 1.53 | 1.52 | 1.66 | 1.68 | 1.84 | 1.88 |
| Brandenburg | 2.31 | 2.39 | 2.34 | 2.36 | 2.27 | 2.46 | 2.60 | 2.59 | 2.57 |
| Mecklenburg-Vorpomern | 2.45 | 2.56 | 2.51 | 2.55 | 2.50 | 2.62 | 2.58 | 2.59 | 2.53 |
| Saxony | 2.35 | 2.43 | 2.50 | 2.47 | 2.34 | 2.49 | 2.53 | 2.48 | 2.51 |
| Saxony-Anhalt | 2.32 | 2.39 | 2.37 | 2.40 | 2.42 | 2.62 | 2.74 | 2.76 | 2.77 |
| Thuringia | 2.37 | 2.34 | 2.50 | 2.64 | 2.54 | 2.57 | 2.62 | 2.49 | 2.54 |

**Online Resource 7: Complications in CLHF patients (2014–2022).** Complications in patients during hospitalization for CHF over the observation period from 2014 to 2022, presented in absolute and relative numbers, differentiated by year. AKI: acute kidney failure, CLHF: Chronic left ventricular heart failure, HTx: heart transplantation, H+LTx: heart and lung transplantation, IABP: intra-aortic balloon pump, LV: left ventricular, ECLS: extracorporeal life support (ECLS).

|  | 2014 | 2015 | 2016 | 2017 | 2018 | 2019 | 2020 | 2021 | 2022 | p-value |
| --- | --- | --- | --- | --- | --- | --- | --- | --- | --- | --- |
|  | % (N) | % (N) | % (N) | % (N) | % (N) | % (N) | % (N) | % (N) | % (N) |  |
| cardiogenic  shock | 1.02% (2,940) | 1.07% (3,135) | 1.1% (3,268) | 1.1% (3,318) | 1.14% (3,328) | 1.18% (3,665) | 1.31% (3,576) | 1.31% (3,656) | 1.3% (3,706) | <0.0001 |
| AKI | 6.47% (18,639) | 8.83% (25,754) | 9.31% (27,556) | 10.1% (30,363) | 10.7% (31,155) | 11.37% (35,442) | 12.33% (33,693) | 13.03% (36,303) | 12.48% (35,634) | <0.0001 |
| renal replacement therapy | 2.09% (6,031) | 2.22% (6,486) | 2.18% (6,443) | 2.24% (6,731) | 2.21% (6,447) | 2.15% (6,704) | 2.43% (6,634) | 2.43% (6,772) | 2.31% (6,591) | <0.0001 |
| resuscitation + LV support | 1.32% (3,815) | 1.4% (4,095) | 1.35% (4,007) | 1.33% (4,007) | 1.37% (3,977) | 1.37% (4,277) | 1.49% (4,072) | 1.45% (4,049) | 1.48% (4,219) | 0.003 |
| Impella, ECLS, IABP | 0.07% (209) | 0.09% (255) | 0.09% (266) | 0.11% (324) | 0.13% (364) | 0.15% (480) | 0.16% (432) | 0.18% (489) | 0.17% (499) | <0.0001 |
| ventilation | 6.26% (18,017) | 6.66% (19,435) | 6.54% (19,367) | 6.61% (19,867) | 6.49% (18,894) | 6.34% (19,755) | 6.46% (17,645) | 6.2% (17,271) | 6.11% (17,429) | <0.0001 |
| univentricular intracorporeal pump | 0.09% (245) | 0.08% (223) | 0.09% (272) | 0.09% (277) | 0.1% (285) | 0.12% (360) | 0.11% (295) | 0.09% (262) | 0.08% (230) | <0.0001 |
| biventricular intracorporeal pump | 0% (0) | 0% (0) | 0% (0) | 0% (0) | 0% (0) | 0% (0) | 0% (0) | 0% (0) | 0% (0) | <0.0001 |
| artificial heart | 0% (0) | 0% (0) | 0% (0) | 0% (0) | 0% (0) | 0% (0) | 0% (0) | 0% (0) | 0% (0) | <0.0001 |
| HTx orthotopic | 0.01% (31) | 0.02% (51) | 0% (0) | 0% (30) | 0% (0) | 0.02% (54) | 0.02% (52) | 0.02% (58) | 0.02% (59) | <0.0001 |
| HTx heterotopic | 0% (0) | 0% (0) | 0% (0) | 0% (0) | 0% (0) | 0% (0) | 0% (0) | 0% (0) | 0% (0) | <0.0001 |
| H+LTx | 0% (0) | 0% (0) | 0% (0) | 0% (0) | 0% (0) | 0% (0) | 0% (0) | 0% (0) | 0% (0) | <0.0001 |
| death | 8.03% (23,137) | 8.54% (24,908) | 7.86% (23,246) | 7.92% (23,786) | 7.87% (22,898) | 7.61% (23,719) | 7.98% (21,819) | 8.27% (23,048) | 8.84% (25,220) | <0.0001 |
